# A novel *DSPP *mutation is associated with type II dentinogenesis Imperfecta in a chinese family

**DOI:** 10.1186/1471-2350-8-52

**Published:** 2007-08-08

**Authors:** Xianqin Zhang, Lanying Chen, Jingyu Liu, Zhen Zhao, Erjun Qu, Xiaotao Wang, Wei Chang, Chengqi Xu, Qing K Wang, Mugen Liu

**Affiliations:** 1 Key Laboratory of Molecular Biophysics of the Ministry of Education, College of Life Science and Technology and Center for Human Genome Research, Huazhong University of Science and Technology, Wuhan, Hubei 430074, China; 2 Department of Bioengineering, Henan Urban Engineering College, Pingdingshan, 467001, China; 3 Department of Molecular Cardiology, Lerner Research Institute, Cleveland Clinic, and Department of Molecular Medicine, Cleveland Clinic Lerner College of Medicine of Case Western Reserve University, Cleveland, Ohio 44195, USA

## Abstract

**Background:**

Hereditary defects of tooth dentin are classified into two main groups: dentin dysplasia (DD) (types I and II) and dentinogenesis imperfecta (DGI) (types I, II, and III). Type II DGI is one of the most common tooth defects with an autosomal dominant mode of inheritance. One disease-causing gene, the dentin sialophosphoprotein (*DSPP*) gene, has been reported for type II DGI.

**Methods:**

In this study, we characterized a four-generation Chinese family with type II DGI that consists of 18 living family members, including 8 affected individuals. Linkage analysis with polymorphic markers *D4S1534 *and *D4S414 *that span the *DSPP *gene showed that the family is linked to *DSPP*. All five exons and exon-intron boundaries of *DSPP *were sequenced in members of type II DGI family.

**Results:**

Direct DNA sequence analysis identified a novel mutation (c.49C→T, p.Pro17Ser) in exon 1 of the *DSPP *gene. The mutation spot, the Pro17 residue, is the second amino acid of the mature DSP protein, and highly conserved during evolution. The mutation was identified in all affected individuals, but not in normal family members and 100 controls.

**Conclusion:**

These results suggest that mutation p.Pro17Ser causes type II DGI in the Chinese family. This study identifies a novel mutation in the DSPP gene, and expands the spectrum of mutations that cause DGI.

## Background

Hereditary defects of tooth dentin include two types of dentin dysplasia (DDI and DDII) and three types of dentinogenesis imperfecta (DGII, II and III). Dentinogenesis imperfecta (DGI) is an autosomal dominant disorder characterized by discolored teeth and often dislodged enamel due to abnormal mineralization of the dentine of the primary teeth [1,2]. The population prevalence rate of DGI is estimated to be 1 in 6,000 to 8,000 individuals [3]. Type I DGI is the least severe and type III is the most severe [1]. Type I DGI is associated with osteogenesis imperfecta, whereas type II DGI and type III DGI are restricted to the dentin.

To date, only one disease-causing gene, *DSPP *encoding the dentin sialophosphoprotein, has been identified for DGI [4]. The *DSPP *gene is located on human chromosome 4q21.3 and transcribed into one transcript which is translated into the DSPP precursor protein [4]. The precursor is cleaved to form two mature proteins, dentin sialoprotein (DSP) and dentin phosphoprotein (DPP). DSP and DPP are the major non-collagenous proteins of dentin expressed in teeth and bone [4]. DPP is rich in aspartic acid and phosphoserine and binds to a large amount of calcium. DSP is a glycoprotein rich in aspartic acid, serine, glutamic acid and glycine [4-7]. Yamakoshi et al. has established that DSP is a proteoglycan, and identified a third domain, the dentin glycoprotein (DGP) domain of DSPP [8,9].

The discovery of *DSPP *as the gene for type II DGI was made in 2001 [10,11]. Several mutations have been reported in *DSPP*. These mutations include five missense mutations (p.Y6D, p.A15V, p.P17T, p.V18F, and p.R68W), one nonsense mutation (p.Q45X), and three splicing mutations (g.1188C→G; g.1275G→A; g.1194C>A (IVS2-3)) [10-16]. Mutation p.Y6D was found to be associated with type II dentin dysplasia (DD) [17], this mutation is located in the signal peptide domain of DSPP, and disabled the entry of DSPP into the endoplasmic reticulum [17]. Mutation p.V18F can cause both type II and type III DGI. Mutation carriers with p.P17V and p.V18F also presented with progressive sensorineural high-frequency hearing loss [11]. The remaining mutations described above cause type II DGI. One *DSPP *mutation was found in a family with type III DGI (a compound mutation consisting of a 36 bp deletion and 18 bp insertion in exon 5) [18].

In this study, we identified and characterized a four-generation Chinese family with type II DGI with 18 living family members and 8 affected individuals. We used linkage analysis to map the disease-causing gene in the family to the *DSPP *gene on chromosome 4q21.3, and further studies identified a novel mutation (c.49C>T, p.Pro17Ser) in *DSPP *in the family. Our finding suggests that Pro17 is a mutational hotspot and it may be critical for the function of *DSPP *gene.

## Methods

### Study subjects and isolation of human genomic DNA

The study participants were identified and enrolled at Luoyang Medical College. Detailed records on medical history, clinical and radiographic features were obtained. Informed written consent was obtained from the study subjects. This study was approved by the ethics committee of Huazhong University of Science and Technology. Peripheral blood was collected from the participants. Human genomic DNA was isolated from the whole blood using the DNA Isolation Kit for Mammalian Blood (Roche Diagnostic Co., Indianapolis, IN).

### Linkage analysis

Two polymorphic microsatellite markers, *D4S414 *and *D4S1534 *linked to *DSPP*, were selected from the ABI PRISM Linkage Mapping Set-MD10 panel for linkage analysis. Markers were genotyped using an ABI 3100 Genetic Analyzer at Huazhong University of Science and Technology Center for Human Genome Research (Applied Biosystems, Foster City, CA). Genotypes were analyzed using the GeneMapper 2 Software program (Applied Biosystems, Foster City, CA).

### Mutation screening

Mutation screening was carried out using direct DNA sequence analysis. All five *DSPP *exons including exon-intron boundaries were PCR-amplified and sequenced. The PCR primers for amplification of the *DSPP *exons and exon-intron boundaries are:

exon 1 forward primer: 5'-TCACCAAGTGAAGGAAGTGG-3'

reverse primer: 5'-AAAGCCCAAGGTGGATTTTT-3'

exon 2 forward primer: 5'-GATGTCCCCATAACCACACC-3'

reverse primer: 5'-CTCCATGACTTCTGGGCATT-3'

exon 3 and 4 forward primer: 5'-CAAGCCCTGTAAGAAGCCACT-3'

reverse primer: 5'-ACATGGATGCTTGTCATGGT-3'

exon 5 forward primer: 5'-CCAGGATGCTTTCAATTACAG-3'

reverse primer: 5'-CCCCCAGTTGTTTTTGTTTA-3'

PCR was performed in 25 μl of standard PCR buffer containing 1.5 mM MgCl_2_, 0.2 mM of each dNTP, 0.5 μM of each primer, 1 unit of Taq DNA polymerase, and 25 ng of human genomic DNA. The amplification program was one cycle of 3 min for denaturation at 94°C, 35 cycles of 30 s at 94°C, 30 s at 65°C, 1 min at 72°C, and one 7 min extension step at 72°C. The PCR products were purified using the QIAquick Gel Extraction Kit (Qiagen Inc., Valencia, CA), and sequenced with both forward and reverse primers. DNA sequencing analysis was performed using the BigDye Terminator Cycle Sequencing v3.1 kit and an ABI PRISM 3100 Genetic Analyzer (Applied Biosystems, Foster City, CA).

### RFLP (restriction fragment length polymorphism) analysis

Mutation c.49C>T(p.Pro17Ser) disrupts a *Bsr I *restriction site, which allowed us to perform RFLP analysis to confirm the mutation and to test whether the mutation co-segregates with the disease in the family. Exon 1 of *DSPP *containing the p.Pro17Ser mutation was PCR-amplified from members of the family as well as 100 unrelated healthy Chinese individuals. The 468 bp PCR product was digested with 1 unit of *Bsr I *restriction enzyme (New England Biolabs Inc, Ipswich, MA) at 65°C for overnight. The digested products were separated and analyzed on a 2.0% agarose gel.

## Results

**Figure 1:**
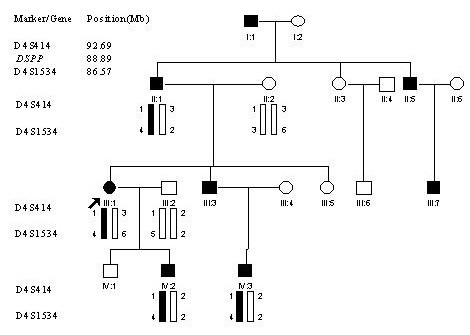
Pedigree structure of a Chinese family affected with type II DGI. Affected males and females are indicated by filled squares and circles, respectively. Normal individuals are shown as empty symbols. The proband is indicated by an arrow. Linkage analysis was performed with two polymorphic microsatellite markers, *D4S414 *and *D4S1534 *linked to the *DSPP *gene. Genotypic results are shown under each symbol. Note that haplotype 1–4 co-segregates with affected individuals, suggesting that the disease-causing gene in the family is linked to *DSPP*.

**Figure 2:**
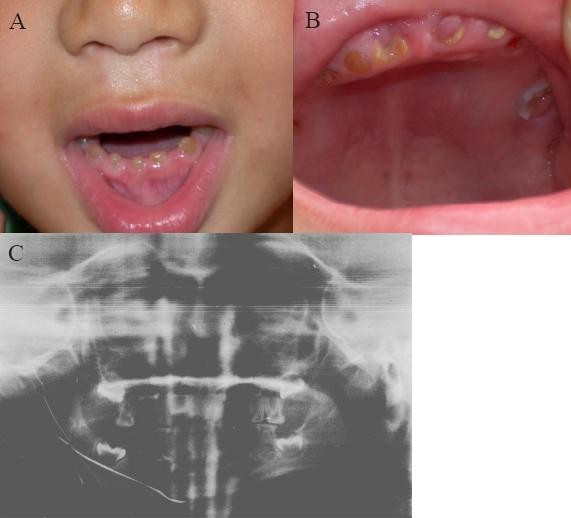
a-b. Oral photographs from the affected individual (IV:2 in Fig. 1). The primary teeth showed shade of brown and almost complete attrition of the enmel layer. c. Panorex radiographs from the affected individual (IV:2 in Fig. 1).

We identified a four-generation Chinese family with type II DGI with 18 living family members and 8 affected individuals. The proband was a 28-year-old female (III-1 in Fig. 1). At the age of 10-years, the enamel was lost through attrition. Similar clinical features were detected in other 7 affected family members. The oral radiographs of the affected son of the proband (IV:2 in Fig. 1) are shown in Fig. 2.

To test whether the *DSPP *gene on chromosome 4q13-21 is the disease-causing gene in the family, we carried out linkage analysis with two markers that span the *DSPP *gene, *D4S414 *and *D4S1534*. As shown in Fig. 1, allele 1 of *D4S414 *and allele 4 of *D4S1534 *co-segregate with the disease in the family. These results suggest that the disease gene in the family is the *DSPP *gene.

**Figure 3:**
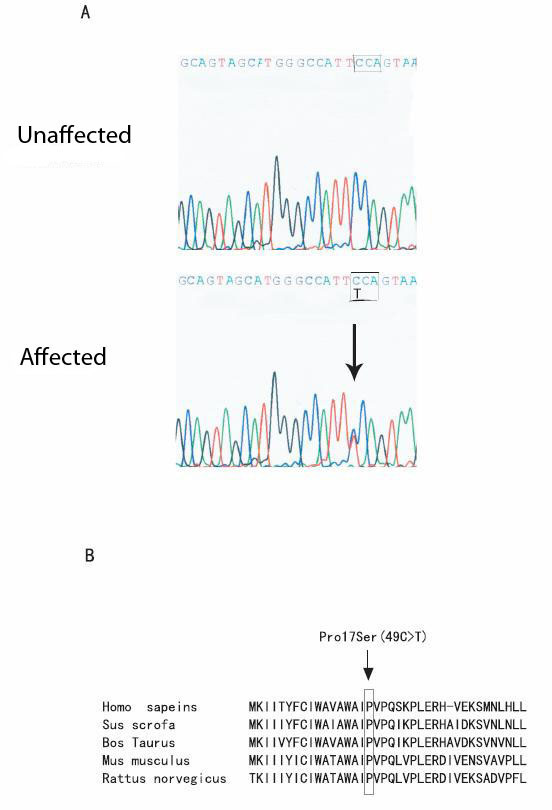
Identification of a novel mutation, g.49C→T/(Pro17Ser), in the *DSPP *gene in the Chinese family with type II DGI. a. DNA sequences for a normal family member (upper panel) and the proband III-1 (lower panel). The sequence of codon 17 where the mutation occurs is boxed. The C to T change in the proband results in the substitution of a proline residue by a serine residue in the DSP protein. The predicted signal peptide domain covers the first 15 amino acids, MKIITYFCIWAVAWA. The mature protein starts with the next isoleucine (I) residue. b. The alignment of amino acids in the N-terminal domain of DSPP from *homo sapiens*, *sus scrofa, bos taurus, mus musculus*, and *rattus norvegious *revealed that the Pro17 residue was highly conserved during evolution.

Direct DNA sequence analysis of the DNA from the proband revealed a heterozygous C→T transition at nucleotide 49 of *DSPP*, which results in a substitution of amino acid residue proline by a serine residue (Pro17Ser) (Fig. 3A). The Pro17 residue of protein DSP is evolutionarily conserved in *homo sapiens*, *rattus norvegicus*, *mus musculus*, *sus scrofa *and *bos taurus*.

**Figure 4:**
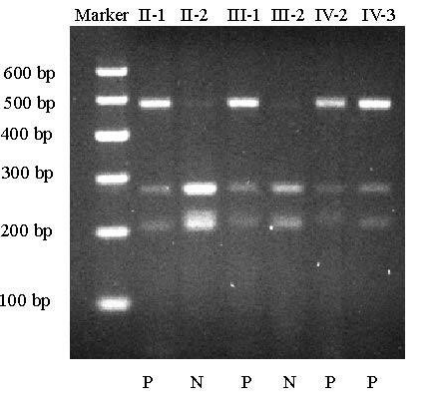
Mutation Pro17Ser of the *DSPP *gene co-segregated with type II DGI in the family. N, normal phenotype; P, affected phenotype; marker, molecular size standard. The lanes are labelled with the unique identification number for each individual in the Chinese family as in Fig. 1. The g.49C>T/Pro17Ser mutation disrupts a *BsrI *restriction site. The wild type PCR product can be cut by *BsrI*, yielding two DNA fragments of 268 bp and 200 bp. The PCR fragment containing mutation Pro17Ser cannot be cut by the enzyme, resulting in only one DNA fragment of 468 bp. All affected individuals in the family are heterozygous for the Pro17Ser mutation (three fragments: 468 bp, 268 bp and 200 bp). Normal family members display two 268 bp and 200 bp bands.

To confirm that the Pro17Ser mutation of *DSPP *is associated with the disease in the family, RFLP analysis was carried out. The patients in the family showed the presence of both wild type allele (268 bp and 200 bp bands) and mutant allele (468 bp) (Fig. 4). RFLP analysis also showed that the p.Pro17Ser mutation was not present in normal family members (Fig. 4) and 100 normal controls (data not shown). These results provide strong genetic evidence that the pPro17Ser mutation of *DSPP *causes type II DGI in the Chinese family.

## Discussion

In this study, we identified a novel mutation, c.49C>T(p.Pro17Ser), in the dentin sialophosphoprotein gene (*DSPP*) in a Chinese family affected with dentinogenesis imperfecta II (type II DGI). The p.Pro17Ser mutation co-segregated with only affected individuals, and did not exist in normal family members and 100 controls. The Pro17 residue is highly conserved during evolution. These results strongly suggest that the p.Pro17Ser mutation causes type II DGI.

Interestingly, the Pro17 residue was mutated to a threonine residue in a different DGI II family with five patients [11]. In contrast to the family in this study, the affected individuals with mutation p.Pro17Thr were also associated with bilateral progressive sensorineural high-frequency hearing loss [11]. Together with the study by Xiao et al., our study suggests that the Pro17 residue is a mutation hot-spot that is critical to the function of *DSPP *gene. Consistent with this conclusion, the Pro17 residue is conserved in *homo sapiens*, *rattus norvegicus*, *mus musculus*, *sus scrofa *and *bos taurus*.

The molecular mechanisms by which *DSPP *mutations cause DGI are not clear. Only one mutation, p.Y6D located in the proposed hydrophobic signal peptide domain, was biochemically characterized. Rajpar et al. showed that the p.Y6D mutation blocked translocation of the proteins encoded by *DSPP *into the endoplamic reticulum(ER), suggesting that it is a loss-of-function mutation [17]. The predicted signal peptide domain of DSP spans the first 15 amino acid residues (MKIITYFCIWAVAWAIPVPQ). The Pro17 residue is only one amino acid residue away from the signal peptide, thus Xiao et al. proposed that the p.Pro17Thr mutation may interfere with signal peptide cleavage [11]. Similarly, the p.Pro17Ser mutation identified in this study may also affect signal peptide cleavage, which may lead to reduced translocation of DSPP to ER. One other possibility is that the p.Pro17Ser mutation may affect pre-mRNA splicing of *DSPP *as it is located at the exon 2/intron 2 boundary, and changes a highly conserved C/A to T at the position of -3 of the 5'-splicing donor site. On the other hand, as the Pro17 residue is the second amino acid residue in the mature DSP protein, it may directly affect the biological functions of DSP. These hypotheses remain to be tested with functional studies.

Knockout mice deficient in *DSPP *showed dentin defects that most closely resemble type III dentinogenesis imperfecta, which has more extreme abnormal dental phenotype that impacts beyond dentin to the enamele and the pulps of affected teeth [19]. The *Dspp*^-/- ^mice displayed the phenotype of enlarged pulp chambers, increased width of predentin zone, hypomineralization, and pulp exposure [19]. Transgenic mice with over-expression of dentin sialoprotein and dentin phosphoprotein were created and characterized [6]. Mice with over-expression of dentin sialoprotein showed an increased rate of enamel mineralization. No significant defects in the enamel morphology were observed. Mice with over-expression of dentin phosphoprotein showed defects in enamel structure with "pitted" and "chalky" enamel of non-uniform thickness [6]. Thus, the knockout mice displayed the phenotype that more closely resemble human DGI than the transgenic overexpression mice, which supports the hypothesis that the *DSPP *mutations may act by a loss-of-function mechanism.

## Conclusion

These results suggest that mutation p.Pro17Ser causes type II DGI in the Chinese family. This study identifies a novel mutation in the DSPP gene, and expands the spectrum of mutations that cause DGI.

## Competing interests

The author(s) declare that they have no competing interests.

## Authors' contributions

XZ carried out genetic studies including linkage and DNA sequence analyses of the *DSPP *gene in affected individuals and controls, RFLP analysis, and drafted the manuscript. LC performed clinical characterization of the family. QKW supervised the study, obtained the funding, and critically revised and approved the manuscript. ML supervised the study and obtained the funding. All other authors provided technical assistance, read and approved the final manuscript.

## Pre-publication history

The pre-publication history for this paper can be accessed here:


